# Therapeutic Drug Monitoring and Pharmacogenetic Study of HIV-Infected Ethnic Chinese Receiving Efavirenz-Containing Antiretroviral Therapy with or without Rifampicin-Based Anti-Tuberculous Therapy

**DOI:** 10.1371/journal.pone.0088497

**Published:** 2014-02-14

**Authors:** Kuan-Yeh Lee, Shu-Wen Lin, Hsin-Yun Sun, Ching-Hua Kuo, Mao-Song Tsai, Bing-Ru Wu, Sue-Yo Tang, Wen-Chun Liu, Sui-Yuan Chang, Chien-Ching Hung

**Affiliations:** 1 Department of Internal Medicine, National Taiwan University Hospital Hsin-Chu Branch, Hsin-Chu, Taiwan; 2 Graduate Institute of Clinical Pharmacy, National Taiwan University, Taipei, Taiwan; 3 Department of Pharmacy, National Taiwan University Hospital and National Taiwan University College of Medicine, Taipei, Taiwan; 4 Department of Internal Medicine, National Taiwan University Hospital and National Taiwan University College of Medicine, Taipei, Taiwan; 5 School of Pharmacy, National Taiwan University, Taipei, Taiwan; 6 Department of Internal Medicine, Far Eastern Memorial Hospital, New Taipei City, Taiwan; 7 Department of Clinical Laboratory Sciences and Medical Biotechnology, National Taiwan University College of Medicine, Taipei, Taiwan; 8 Department of Laboratory Medicine, National Taiwan University Hospital and National Taiwan University College of Medicine, Taipei, Taiwan; 9 Department of Medical Research, China Medical University Hospital, Taichung, Taiwan; 10 China Medical University, Taichung, Taiwan; CEA, France

## Abstract

**Objectives:**

Plasma efavirenz concentrations in HIV-infected patients with tuberculosis (TB) may be affected by cytochrome P450 (CYP) 2B6 single-nucleotide polymorphisms and concurrent rifampicin use. We aimed to investigate the effects of *CYP2B6* G516T polymorphisms and concomitant rifampicin use on the plasma efavirenz concentrations in HIV-infected Taiwanese.

**Methods:**

HIV-infected patients with or without TB who had received combination antiretroviral therapy containing efavirenz (600 mg daily) for two weeks or greater were enrolled for determinations of *CYP2B6* G516T polymorphism and plasma efavirenz concentrations with the use of polymerase-chain-reaction restriction fragment-length polymorphism and high-performance liquid chromatography, respectively.

**Results:**

From October 2009 to August 2012, 171 HIV-infected patients, including 18 with TB, were enrolled 113 (66.1%) with *CYP2B6* G516G, 55 (32.2%) GT, and 3 (1.8%) TT genotype. Patients receiving rifampicin had a significantly lower median plasma efavirenz concentration than the control group (2.16 vs 2.92 mg/L, *P* = 0.003); however, all patients achieved target plasma concentration (>1 mg/L). Patients with GT or TT genotype had a significantly higher plasma concentration than those with GG genotype (2.50 vs 3.47 mg/L for GT genotype and 8.78 mg/L for TT genotype, *P*<0.001). Plasma efavirenz concentration >4 mg/L was noted in 38 (22.2%) patients, which was associated with a lower weight (per 10-kg increase, odds ratio, 0.52; 95% confidence interval, 0.33–0.83) and GT or TT genotype (odds ratio, 4.35; 95% confidence interval, 1.97–9.59) in multivariate analysis.

**Conclusions:**

Despite combination with rifampicin, sufficient plasma efavirenz concentrations can be achieved in HIV-infected Taiwanese with TB who receive efavirenz 600 mg daily. Carriage of *CYP2B6* 516 GT and TT genotypes and a lower weight are associated with higher plasma efavirenz concentrations.

## Introduction

Efavirenz is a potent non-nucleoside reverse-transcriptase inhibitor (NNRTI) and its combination with tenofovir and emtricitabine remains one of the preferred antiretroviral regimens for antiretroviral-naive patients without resistance-associated mutations to NNRTIs [1]. The recommended therapeutic levels of efavirenz at 12 hours are 1 to 4 mg/L [2]. Sub-therapeutic drug concentrations may increase the risk of drug resistance and treatment failure; on the other hand, concentrations above the therapeutic range may increase the risk of drug-related toxicities, such as neuropsychiatric side effects, which may lead to emergence of resistance resulting from treatment interruptions [2].

Efavirenz is metabolized primarily through hepatic cytochrome P450 (CYP) 2B6. The wide inter-patient variability of efavirenz concentrations has been reported, which may be related to sex, weight, ethnicity, drug-drug interactions, and single-nucleotide polymorphism (SNP) of *CYP2B6* [3]–[8]. However, conflicting data regarding the aforementioned factors makes dose adjustment while co-administration with other drugs or weight-based adjustment problematic [9], [10].

Efavirenz-containing antiretroviral therapy has been recommended for HIV-infected patients with tuberculosis (TB) when rifampicin-containing anti-tuberculous therapy is co-administered. Rifampicin may induce activity of CYP enzymes, which may lower the plasma concentrations of efavirenz. The early pharmacokinetic studies reported a reduction by 26% in the efavirenz concentration when co-administered with rifampicin [11], and the package insert of efavirenz recommends a compensatory increase of efavirenz from a standard dose of 600 mg to 800 mg per day for patients who are taking concomitant rifampicin and have a weight greater than 50 kg [9], [12]. However, a recently published clinical trial demonstrated the opposite findings and did not support weight-based dosing of efavirenz in combination with rifampicin [10]. In addition, prior studies have shown that *CYP2B6* polymorphism, particularly G516T, is associated with high plasma concentrations of efavirenz and its drug-related toxicity [7], [13], [14]. The frequency of *CYP2B6* G516T polymorphisms may vary with different racial populations [15], and the pharmacogenetic data of efavirenz concentrations in Taiwanese patients are lacking. In this study, we aimed to investigate the effect of concurrent use of rifampicin and *CYP2B6* G516T polymorphisms on the plasma efavirenz concentrations in HIV-infected Taiwanese patients.

## Materials and Methods

### Study Population

From October 2009 to August 2012, HIV-infected patients who were aged 18 years or greater and had been receiving an efavirenz-containing combination antiretroviral therapy at a daily dose of 600 mg for more than 14 days were enrolled when they sought routine HIV care at the National Taiwan University Hospital, the largest referral hospital for inpatient and outpatient HIV care in Taiwan. For patients who received a clinical or microbiologically confirmed diagnosis of TB, the daily dose of rifampicin was 450 mg for those with a weight less than 50 kg and 600 mg for those with a weight of 50 kg or greater. Patients were excluded from the study if they were pregnant; infected with HIV that was shown to harbor resistance-associated mutations to efavirenz or other NNRTIs; receiving anti-tuberculous regimens that did not contain rifampicin; infected with rifampicin-resistant *Mycobacterium tuberculosis*; or had hepatic transaminases greater than five times the upper limit of normal. The protocol was approved by the Research Ethics Committee of the National Taiwan University Hospital (registration number, 200908014M) and all patients provided written informed consent prior to enrollment.

### Data Collection and Sample Preparation

We used a computerized case record form to collect data on the demographics, weight and height, and clinical characteristics including CD4 lymphocyte counts and plasma HIV RNA loads at baseline and during the follow-up, serostatus of hepatitis B virus and hepatitis C virus, concomitant medications (including antiretroviral therapy and rifampicin), and the date and time of the last efavirenz dose that was usually taken at bedtime. Blood samples were collected 12±1 hours after the last dose of efavirenz into tubes containing EDTA as anticoagulant for *CYP2B6* G516T genotyping and determination of efavirenz concentration. Plasma samples were stored at −20°C until analysis.

### Laboratory Investigations

#### Determination of plasma HIV RNA load and CD4 lymphocyte count

Plasma HIV RNA load was quantified using the Cobas Amplicor HIV-1 Monitor test (Cobas Amplicor version 1.5, Roche Diagnostics Corporation, IN) with a lower detection limit of 40 copies/mL, and CD4 lymphocyte count was determined using FACFlow (BD FACS Calibur, Becton Dickinson, CA). The CD4 counts and plasma HIV RNA loads were monitored one month after initiation of combination antiretroviral therapy in antiretroviral-naive patients, or change of regimens in the presence of virological failure; and every three to six months thereafter according to the national HIV treatment guidelines.

#### *CYP2B6* G516T genotyping

High molecular weight genomic DNA was extracted from PBMC using the Wizard® Genomic DNA purification kit (Promega, WI, USA). The concentration of extracted DNA was determined by spectrophotometry and stored at −20°C before further analysis. Polymerase-chain-reaction restriction fragment-length polymorphism (PCR-RFLP) was performed to determine the SNPs of *CYP2B6* G516T.

#### Determination of efavirenz concentrations

The plasma concentration of efavirenz was analyzed using high-performance liquid chromatography (HPLC) based on a validated method reported by Ramachandran et al. [16] with minor modifications. In brief, 300 µL of plasma was added to 300 µL of acetonitrile for deproteinization, and the organic layer was dried under nitrogen. The extract was then dissolved with 150 µL of methanol and 200 µL of 10 mM phosphate buffer (pH 4.0) and acetonitrile in a 57∶43 (volume/volume) ratio. The HPLC system consisted of a L-2130 HTA solvent delivery pump, a L-2200 autosampler, a UV1000 wavelength detector programmable UV detector wavelength 245 nm and the computing integrator for HPLC D-2000 Elite on Windows (version 1.2, Hitachi High Technologies Corporation, Tokyo, Japan). Chromatography was performed on a C18 column (Mightsil RP-18 GP, 250×4.6 nm with 5 µm beads, particle size 3.5 mm; Kanto Corporation, Portland, OR, USA) protected by a guard column (1033 mm I.D.; Phenomenex, Torrance, CA, USA), and a flow rate of 1 mL/min. The mobile phase was composed of the same phosphate buffer mixed with acetonitrile (57∶43). The retention time of efavirenz was 13.43 min. The calibration curve was linear within the range 0.5 to 10.0 mg/L, and data more than 10.0 mg/L was designated as 10.0 mg/L. The lower limit of quantification was 0.5 mg/L. Recovery after extraction from plasma was 101%. Accuracy ranged from 97.7 to 101.6%. Intra-assay and inter-assay coefficients of variation at 5 µg/mL ranged from 0.2 to 0.97% and 1.15 to 1.93%, respectively.

### Statistical Analysis

Statistical analysis was performed using SPSS software (version 17.0; SPSS Inc., Chicago, IL, USA). Continuous variables were reported as medians and ranges, and were compared using the Mann-Whitney test. Categorical variables were expressed as numbers and percentages, and were compared using the Pearson χ2 test or Fisher’s exact test, as appropriate. Multivariate logistic regression analysis was used to evaluate the association between each independent variable and risk of high plasma efavirenz concentration (>4 mg/L). Variables with *P* values <0.10 in the univariate analyses were entered into a multivariate logistic regression model with the backward elimination method. Pearson’s correlations were used to evaluate the relationships between body weight and plasma efavirenz concentration. *P* values <0.05 were considered statistically significant.

## Results

During the study period, 171 HIV-infected patients, all of whom were ethnic Chinese, were enrolled. The baseline characteristics of the patients are shown in Table 1. Most of the patients were male (93.6%) with a median age of 38 years (range, 19–87 years). The median weight was 62 kg (range, 40.5–95 kg). About three quarters of the patients (126, 73.7%) had been receiving combination antiretroviral therapy for 24 weeks or more with a plasma HIV RNA load <200 copies/mL in 121 (96.0%). The median CD4 count was 401 cells/µL (range, 3–1377 cells/µL). Eighteen patients (10.5%) were also taking rifampicin-containing anti-tuberculous therapy, including 15 with microbiologically-confirmed TB, two with subsequently microbiologically-confirmed non-tuberculous mycobacteriosis, and one with TB diagnosed based on clinical presentation. Seventy-seven (45.0%) patients received concomitant medications other than antiretroviral and anti-tuberculous therapy, and none of these drugs had been reported to cause significant drug-drug interaction with efavirenz.

**Table 1:** Clinical characteristics of 171 HIV-infected patients who received efavirenz-containing combination antiretroviral therapy with or without receiving concurrent rifampicin-containing anti-tuberculous therapy.

		Concurrent Rifampicin Use				*CYP2B6* G516T Polymorphisms		
	All patients(n = 171)	Rifampicin (n = 18)	Control (n = 153)	P value	GG (n = 113)	GT (n = 55)	TT (n = 3)	P value
Age, median (range), years	38 (19–87)	44 (24–69)	37 (19–87)	0.08	38 (19–87)	38 (21–72)	37 (31–38)	0.74
Male sex, n (%)	160 (93.6)	18 (100)	142 (92.8)	0.61	105 (92.9)	52 (94.5)	3 (100)	>0.99
Homosexual male, n (%)	136 (79.5)	16 (88.9)	120 (78.4)	0.48	86 (76.1)	47 (85.5)	3 (100)	0.31
Body weight, kg, median (range)[patients with available data]	62 (40.5–95) [170]	59 (41–73) [18]	62 (40.5–95) [152]	0.040	62 (40.5–95) [113]	62 (47–81) [54]	61 (61–65) [3]	0.97
Body-mass index, median (range),kg/m^2^ [patients with available data]	21.5 (14.5–29.8) [167]	20.7 (14.5–25.0) [18]	21.5 (15.4–29.8) [149]	0.26	21.9 (14.5–21.8) [110]	21.3 (17.5–29.7) [54]	21.0 (20.1–21.2) [3]	0.69
CART-naive, n (%)	45 (26.3)	9 (50.0)	36 (23.5)	0.041	26 (23.0)	18 (32.7)	1 (33.3)	0.39
Plasma HIV viral load at baseline, median (range), log_10_ copies/mL	1.6 (1.6–6.3)	4.2 (1.6–5.8)	1.6 (1.6–6.3)	<0.001	1.6 (1.6–6.1)	1.6 (1.6–6.3)	1.6 (1.6–4.8)	0.57
Plasma HIV viral load <200 copies/mL at baseline, n (%)	121 (70.8)	6 (33.3)	115 (75.2)	<0.001	83 (73.5)	36 (65.5)	2 (66.7)	0.56
CD4 at baseline, median (range), cells/µL	401 (3–1377)	119 (3–1100)	413 (11–1377)	0.001	430 (3–1377)	358 (14–1037)	300 (232–586)	0.33
Chronic HBV infection, n (%)	37 (21.6)	3 (16.7)	34 (22.2)	0.77	20 (17.7)	17 (30.9)	0 (0)	0.12
Chronic HCV infection, n (%)	14 (8.2)	3 (16.7)	11 (7.2)	0.17	8 (7.1)	6 (10.9)	0 (0)	0.53
Total bilirubin, median (range), mg/dL[patients with available data]	0.51 (0.13–5.64)[161]	0.94 (0.2–3.93) [16]	0.49 (0.13–5.64) [145]	0.001	0.53 (0.13–3.17) [108]	0.48 (0.21–5.64) [50]	0.41 (0.34–0.57) [3]	0.64
AST, median (range), U/L [patients with available data]	25 (12–241) [163]	37 (15–76) [18]	24 (12–241) [145]	0.07	24 (12–241) [109]	25 (12–135) [51]	32 (24–42) [3]	0.57
*CYP2B6* G516T polymorphisms, n (%)								
GG	113 (66.0)	12 (66.7)	101 (66.0)	0.84				
GT	55 (32.2)	6 (33.3)	49 (32.0)	0.88				
TT	3 (1.8)	0 (0)	3 (2.0)	>0.99				
Concurrent rifampicin use, n (%)					12 (10.6)	6 (10.9)	0 (0)	>0.99

* Abbreviations: AST, aspartate aminotransferase; cART, combination antiretroviral therapy; HBV, hepatitis B virus; HCV, hepatitis C viru.

All 171 patients had determinations of *CYP2B6* G516T polymorphism: 113 patients (66.1%) had carriage of GG genotype (wild-type), 55 (32.2%) GT genotype (heterozygous mutant), and 3 (1.8%) TT genotype (homozygous mutant). Compared with those who did not have TB, patients with TB and receiving rifampicin had a lower weight (median, 59 vs 62 kg, *P* = 0.040), though the body-mass index (BMI) between the two groups was similar (Table 1); were less likely to have had combination antiretroviral therapy before enrollment into this study (50.0% vs 76.5%, *P* = 0.041); and were more likely to have higher levels of total bilirubin levels in the serum (median, 0.94 vs 0.49 mg/dL, *P* = 0.001). The demographics, HIV-related variables, and laboratory data were all similar among the patients with different genotypes of *CYP2B6* G516T (Table 1).

**Figure 1:**
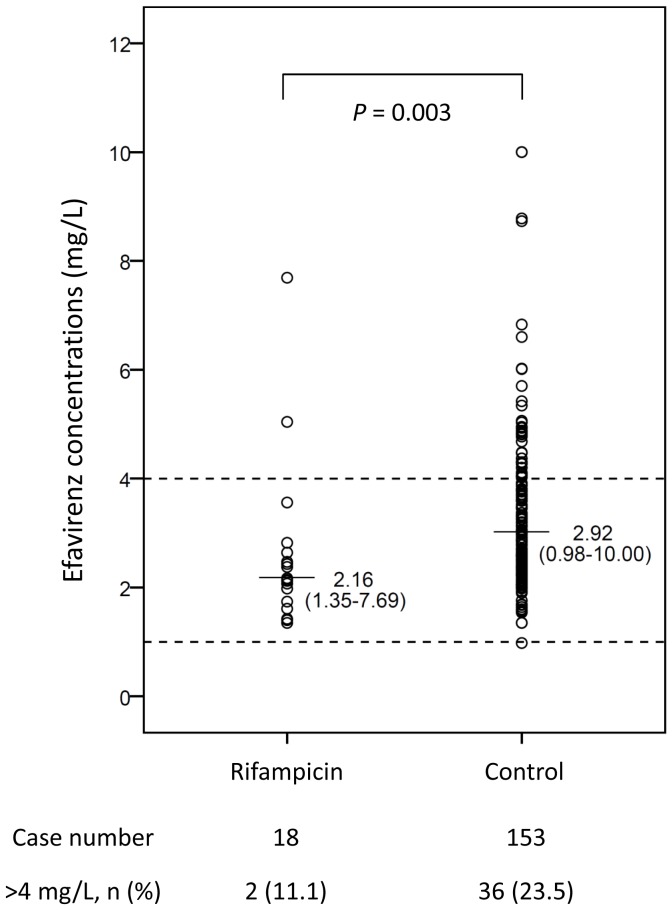
Plasma efavirenz concentrations of HIV-infected patients with (n = 18) and without taking rifampicin (n = 153). Dash lines indicate the target plasma concentrations of efavirenz for wild-type HIV-1 isolate.

**Figure 2:**
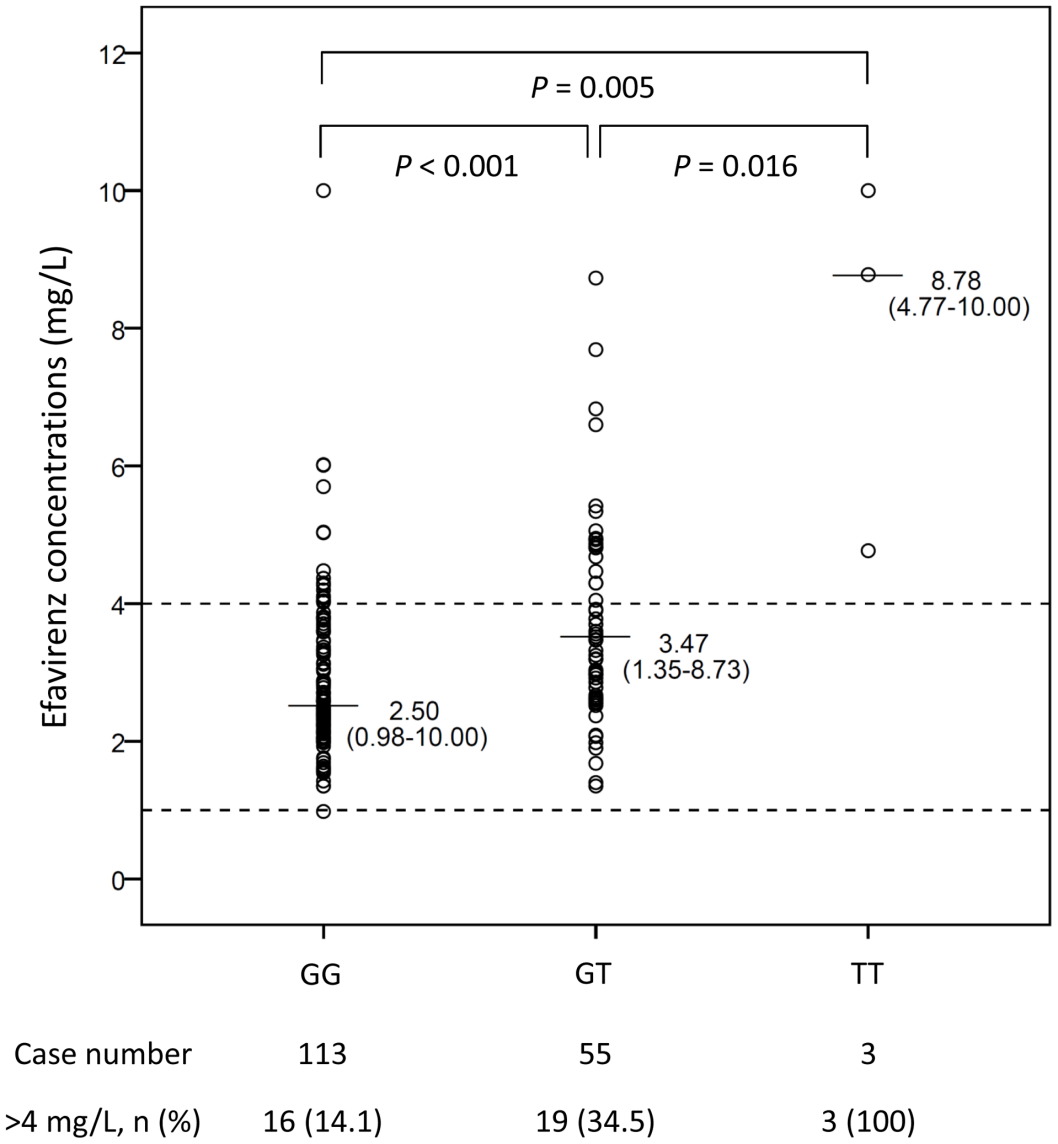
Plasma efavirenz concentrations of 171 HIV-infected patients with different genotypes of *CYP2B6* G516T polymorphisms. Dash lines indicate the target plasma concentrations of efavirenz for wild-type HIV-1 isolate.

The median plasma concentration of efavirenz at 12 hours for all patients was 2.82 mg/L (range, 0.98–10.00 mg/L), and almost all of the patients achieved plasma efavirenz concentration of more than 1 mg/L except one with a level of 0.98 mg/L. Figure 1 shows the comparison of plasma efavirenz concentrations between the patients with and those without receiving rifampicin. Compared with the patients not receiving rifampicin, patients receiving rifampicin had a significantly lower median plasma concentration by 26% (median [range], 2.16 [1.35–7.69] vs 2.92 [0.98–10.00] mg/L, *P* = 0.003). Nevertheless, all patients receiving rifampicin achieved plasma efavirenz concentrations above the recommended target concentration (>1 mg/L), even in the eight patients who weighted 60 kg or more (the plasma concentrations ranging from 1.35 to 2.82 mg/L). Figure 2 shows the plasma efavirenz concentrations in patients with different *CYP2B6* G516T genotypes. Patients with heterozygous or homozygous mutants had significantly higher plasma efavirenz concentrations compared to those with wild type (median [range], 2.50 mg/L [0.98–10.00] for GG genotype vs 3.47 mg/L [1.35–8.73] for GT genotype and 8.78 mg/L [4.77–10.00] for TT genotype; *P*<0.001).

**Figure 3:**
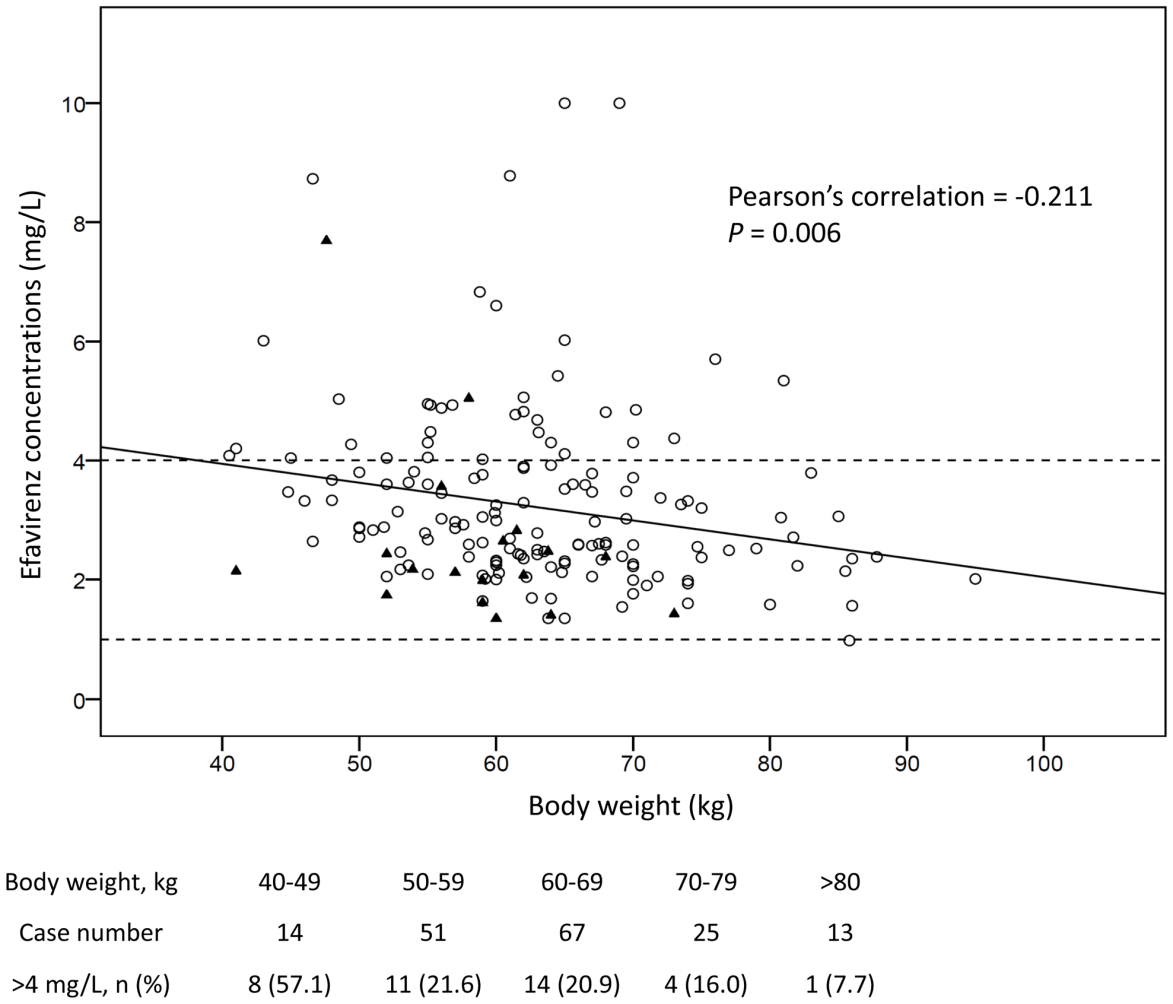
Relationship between efavirenz plasma concentrations (mg/L) and body weight (kg). Triangles indicate cases with concurrent rifampicin use, and circles indicate cases without taking rifampicin.

More than one fifth of the patients (38, 22.2%) had plasma efavirenz concentrations greater than 4 mg/L, a concentration that was reported to be associated with a higher risk for neuropsychiatric adverse effects [2]. Univariate and multivariate analyses revealed that a lower weight and heterozygous or homozygous mutant of *CYP2B6* G516T genotypes were statistically significantly associated with elevated plasma efavirenz concentrations above 4 mg/L (Table 2). Figure 3 shows that plasma efavirenz concentrations were inversely correlated with weight, and leaner patients (especially those <50 kg) had plasma efavirenz concentrations greater than 4 mg/L.

**Table 2:** Univariate and multivariate logistic regression analyses of variables associated with toxic plasma efavirenz concentrations (>4 mg/L).

	Univariate analysis		Multivariate analysis	
	OR (95% CI)	*P* value	OR (95% CI)	*P* value
Age, years	0.98 (0.95, 1.02)	0.36		
Body weight, per 10-kg increase	0.59 (0.39, 0.89)	**0.011**	0.52 (0.33, 0.83)	**0.006**
Chronic HBV infection	0.96 (0.40, 2.31)	0.92		
Chronic HCV infection	0.56 (0.12, 2.62)	0.46		
Rifampicin use	0.41 (0.09, 1.85)	0.24		
*CYP2B6* 516 GT or TT genotypes	3.71 (1.75, 7.84)	**0.001**	4.35 (1.97, 9.59)	**<0.001**

Abbreviations: 95% CI, 95% confidence interval; OR, odds ratio.

Fourteen of 18 patients (77.8%) receiving rifampicin and 143 of 153 patients (93.5%) not receiving rifampicin continued efavirenz-containing combination antiretroviral therapy for at least 24 weeks. Among the patients receiving rifampicin, three died of bacterial pneumonia complicated with respiratory failure and one was transferred to another hospital. On the other hand, among the patients not receiving rifampicin, one patient died of bacterial pneumonia complicated with respiratory failure; two discontinued efavirenz due to intolerable neuropsychiatric side effects with plasma efavirenz concentrations of 4.08 and >10.0 mg/L, respectively; three changed antiretroviral regimen because of genotypic resistance to efavirenz; and the other four were lost to follow-up. The proportion of patients with successful virological suppression (plasma HIV RNA load <200 copies/mL) at week 24 was similar between the patients receiving rifampicin and those not receiving rifampicin (92.9% vs 99.3%, *P* = 0.34). As for the 45 antiretroviral-naïve patients, 8 and 32 patients in each group continued efavirenz-containing combination antiretroviral therapy for at least 24 weeks, and the proportions of patients who achieved virological suppression was also similar (87.5% vs 96.9%, *P* = 0.73).

## Discussion

This therapeutic drug monitoring and pharmacogenetic study that was performed in HIV-infected Taiwanese individuals showed that almost all patients could achieve therapeutic efavirenz concentrations (>1 mg/L) regardless of *CYP2B6* G516T polymorphism, rifampicin use, or body weight. In contrast, a significant proportion of the patients had toxic levels (>4 mg/L), which was statistically significantly associated with a lower weight and GT or TT genotypes of *CYP2B6* G516T.

Efavirenz-containing combination antiretroviral therapy is the preferred antiretroviral therapy for HIV-related TB for which rifampicin-based anti-tuberculous therapy is co-administered [1]. However, data are conflicting on the appropriate efavirenz dose when co-administered with rifampicin among the published studies [7], [10]–[12], [17], [18]. The discrepancy may be related to the study populations with different body habitus and pharmacogenetics. Early studies reported a 26% reduction in the plasma efavirenz concentration when co-administered with rifampicin [11], [12], and the possible mechanism is that rifampicin induces the activity of the main metabolic enzyme, CYP2B6, which results in enhanced efavirenz clearance and reduced plasma levels. As a result, the US Food and Drug Administration approved a revised efavirenz package insert recommending efavirenz be increased from a standard daily dose of 600 mg to 800 mg for patients taking concomitant rifampicin who weigh greater than 50 kg [9], based on empirical data from the two drug-drug interaction trials [11], [12] and semi-mechanistic population pharmacokinetic modeling.

In contrast, more recent and larger studies in HIV-infected patients with TB indicated either that there was no significant impact of rifampicin on efavirenz concentrations [7], [17]; or that rifampicin co-administration increased efavirenz concentrations [10], [18]. The study by Ramachandran et al. conducted in South India [7] and another study by Cohen et al. in South Africa (with a mean weight of 66 kg among participants) [17] both demonstrated that *CYP2B6* G516T polymorphism but not rifampicin co-administration significantly influenced the pharmacokinetics of efavirenz. The study by Kwara et al. found that patients receiving rifampicin had significantly higher mean efavirenz concentrations in those with the *CYP2B6* 516TT genotype [18]. The authors hypothesized that the paradoxical effect may be due to increased susceptibility of the CYP2B6 (172-histidine) variant allozyme (resulting from the 516G→T polymorphism) to inhibition by one (or more) of the anti-tuberculous drugs as compared with the reference CYP2B6 (172-glutamine) enzyme, and metabolic inhibition on the non-CYP2B6 accessory pathways, including CYP2A6 by isoniazid. More studies are warranted to determine the underlying mechanism. The large multinational and multicenter STRIDE Study that enrolled 543 participants showed that efavirenz and rifampicin co-administration was associated with a trend toward higher, not lower, efavirenz trough concentrations compared to efavirenz alone in all patients (median, 1.96 vs 1.80 mg/L, *P* = 0.067), and the concentrations were significantly higher in blacks (median, 2.08 vs 1.75 mg/L, *P* = 0.005) [10]. CYP2B6 genotyping for the participants of the STRIDE pharmacokinetic study is planned for further analysis to determine whether the CYP2B6 genetic polymorphisms play an important role in the paradoxical increases in efavirenz concentrations with rifampicin co-administration.

In our study, patients taking rifampicin had significantly lower plasma efavirenz concentrations compared to the patients not receiving rifampicin, but all of them could achieve plasma efavirenz concentrations above the therapeutic target. Besides, most of the patients (88.9%) weighed greater than 50 kg. As a result, our findings suggest that the standard daily 600 mg efavirenz dose is adequate for patients on efavirenz-containing combination antiretroviral therapy who receive concurrent rifampicin for the treatment of TB. Of note, there were only three patients with the *CYP2B6* 516TT genotype, and none of them were taking rifampicin concomitantly in our study. Therefore, more studies are needed to determine if the same phenomenon of paradoxically elevated efavirenz concentrations in patients with *CYP2B6* 516TT genotype receiving rifampicin will be seen in Chinese population.

Efavirenz is mainly metabolized by CYP2B6, and the *CYP2B6* gene is highly polymorphic. Many previous studies have shown *CYP2B6* SNP, particularly G516T, to be associated with higher plasma efavirenz concentrations and its drug-related toxicity, which could result in early discontinuation of antiretroviral therapy [19], [20]. The allele frequency of *CYP2B6* G516T varies among the different ethnicities, ranging from 14% to 21% in Asian populations [15], [21], [22], 22% to 30% in Caucasians [8], [20], [23], and up to near 50% in Africans [13], [24]. In our study, the frequency of *CYP2B6* G516T was 17.8% (32.2% of GT genotype and 1.8% of TT genotype), which was similar to those in prior Asian studies [15], [21], [22]. To avoid high plasma efavirenz concentrations and related drug toxicities, therapeutic drug monitoring and pharmacogenetic-guided dosing strategy that have been described by Gatanaga et al. [25] would be helpful and should be introduced into clinical practice.

Several studies have demonstrated that a higher weight were associated with lower efavirenz levels [5], [6], but how to adjust doses according to the weight remains a debatable issue. Some studies suggested an increase of efavirenz dose for heavy patients taking rifampicin [26], while other studies showed data arguing against this suggestion [10], [27]. In our study, nearly all patients could achieve therapeutic concentrations, and a significant proportion of the patients who had a lower weight achieved plasma efavirenz concentrations of greater than 4 mg/L (figure 3). As a result, a reduced dose of efavirenz with the information of therapeutic drug monitoring will be beneficial to Taiwanese patients with a lower weight to minimize the risk of adverse events and save treatment costs.

Our study has several limitations. First, it was performed at a single hospital in Taiwan, with most patients being male. Information on the efavirenz concentrations among HIV-infected female patients remains limited. Second, only patients who could tolerate efavirenz for two weeks or more were included in this study, and three quarters of the patients had been receiving efavirenz for more than 24 weeks. Therefore, the proportion of patients with toxic drug levels may be underestimated since patients with significantly higher drug concentrations may have interrupted or changed regimen earlier than two weeks because of severe psychiatric side effects and skin rashes. Third, this was a cross-sectional study, and we did not collect serial blood samples when the patients received and after they discontinued rifampicin-containing anti-tuberculous therapy, to avoid inter-patient variations. Last, we did not collect information on neuropsychiatric adverse effects to correlate with plasma efavirenz concentrations.

In conclusion, with information obtained from therapeutic drug monitoring and pharmacogenetic study in HIV-infected Taiwanese, we have shown that most patients could achieve therapeutic efavirenz concentrations, with or without combination with rifampicin, when receiving a standard 600 mg of efavirenz daily; and lower weights and *CYP2B6* 516 GT and TT genotypes were associated with higher plasma efavirenz concentrations that might be associated with an increased risk of neuropsychiatric adverse effects.
